# Use of implanted acoustic tags to assess platypus movement behaviour across spatial and temporal scales

**DOI:** 10.1038/s41598-018-23461-9

**Published:** 2018-03-23

**Authors:** Gilad Bino, Richard T. Kingsford, Tom Grant, Matthew D. Taylor, Larry Vogelnest

**Affiliations:** 1 Centre for Ecosystem Science, School of Biological, Earth & Environmental Sciences, UNSW Australia, Sydney, 2052 NSW Australia; 2 Port Stephens Fisheries Institute, NSW Department of Primary Industries – Fisheries, Taylors Beach Rd, Taylors Beach, New South Wales, 2316 Australia; 3 Taronga Conservation Society, Bradleys Head Rd, Mosman, 2088 NSW Australia

## Abstract

The platypus (*Ornithorhynchus anatinus*) is an evolutionarily distinct mammal, endemic to Australian freshwaters. Many aspects of its ecology and life-history, including detailed understanding of movements, are poorly known, hampered by its cryptic and mainly nocturnal habits and small numbers. We effectively trialled intraperitoneal implanted acoustic transmitters in nine platypuses in the Severn River (NSW), Australia, as a potential approach for studying movements in this challenging species. We tracked platypus movements over six months, at fine and broad spatial scales, using an array of acoustic sensors. Over six months (March-August 2016), four of five adult platypuses (two females\three males) maintained localized movements (average monthly maximums 0.37 km ± 0.03 sd), while one adult, one sub-adult, and one juvenile (males) moved further: average monthly maxima 1.2 km ± 2.0 sd, 0.9 km ± 0.6 sd, 4.5 km ± 5.9 sd, respectively. The longest recorded movement was by a male adult, covering 11.1 km in three days and travelling a maximum distance of about 13 km between records. Only one implanted animal was not detected immediately after release, indicative of transmission failure rather than an adverse event. High cumulative daily movements (daily 1.9 km ± 0.8 sd) indicated high metabolic requirements, with implications for previous estimates of platypus abundances and carrying capacities, essential for effective conservation. This novel approach offers new avenues to investigate relating to mating, nesting, and intraspecific competition behaviours and their temporal and spatial variation.

## Introduction

The platypus (*Ornithorhynchus anatinus*) is one of only five extant species of egg-laying mammals, a unique evolutionarily distinct mammal^1–3^. It is considered ubiquitous although seldom numerous, occurring along the margins of the eastern Australian mainland, in Tasmania and adjacent King Island, with a small introduced population on Kangaroo Island. Platypuses live and breed mainly in permanent reaches of streams and in some lakes and wetlands, from which most juveniles\sub-adult disperse in their first year of life^4^. Although knowledge of platypus biology and life-history is increasing^5–7^, the animal’s secretive and aquatic nature, mainly nocturnal habits, and small numbers have made it a challenging species to research, particularly its movement behaviour. Secretive and elusive species have challenged researchers to develop technology and to study and monitor populations^8^. Technological progress in telemetry devices have enabled tracking of movement behaviour and physiological states for an ever-expanding range of species over five decades^9^, with recent advances in data-loggers and transmitters offering insights into the ecology and behaviour of many aquatic species^10^.

Current understanding of platypus movements has primarily come from capture/recapture studies, observations, radio-tracking, microchip implantation and externally-attached acoustic tags, revealing considerable variability among individual movements, varying with sex, location, season and timing of breeding^4,11^. Mark-recapture studies have identified linear home ranges up to 4 km in females and 4–14 km in males, with limited information about juvenile dispersal and establishment^12,13^. Unfortunately, mark-recapture rates are consistently low and require significant effort, constraining understanding, particularly of fine scale movements, even with intensive marking of animals^5,13^. Radio tracking has revealed movement ranges of 400m-15.1 km over a few weeks, with males travelling linear distance of as much as 10.4 km overnight^11,14–16^. Short term (up to 2 months) radio-tracking and activity loggers, show activity periods of 10–12 hours, with increased activity in winter^14,16–18^. Unlike other freshwater mammals such as Eurasian otters (*Lutra lutra*)^19^ or beavers (*Castor canadensis*)^20^, access to fine scale data using telemetry (GPS, radio or acoustic) are particularly constrained due to the impracticality of attaching a collar or harness to platypuses because of a high risk of strangulation or suffocation underwater as the animals use their bill to forage between submerged roots and branches and dig their burrows between tree roots^4^. Gluing of radio and acoustic trackers overcomes some of these problems but has short attachment periods (average 24 days, range 0–51, n = 24), due to fur growth resulting in detachment^21,22^. Sub-cutaneous injected Passive Integrated Transponder tags (‘microchips’) were used to track platypuses in Tasmania over approximately two years, potentially extending indefinitely as these do not require batteries, but are extremely limited by short detection distances (<1 m), constraining application to small streams and recaptures^23^. Implanted telemetry devices (subcutaneous, intraperitoneal) have been successfully used in many similar aquatic mammals to platypus, including North America river otters (*Lontra canadensis*)^24^, Eurasian otters (*Lutra lutra*)^25^, muskrats (*Ondatra zibethicus*)^26^, beavers (*Castor canadensis*)^27^, and nutrias (*Myocastor coypus*)^28^. In platypus, intraperitoneally implanted radio-telemetry units provided body temperature records for five free-ranging-platypuses during winter in the Thredbo River in NSW, but resultant data were limited by transmitter failure, with only two individuals monitored over 6 months, autumn to spring^29^. With the development of smaller and more sophisticated tracking and data-logging devices, including radio and acoustic tags, understanding of the behaviour and life history of the platypus in the wild has improved offering better understanding of movement behaviour.

We trialled the effectiveness of intraperitoneal implanted acoustic transmitters in platypuses, using an array of acoustic sensors to track movements over six months and avoiding constraints of poor attachment periods. This allowed us to investigate detailed fine and large-scale movements and habitat use. We examined movement behaviours, aiming to quantify ranges, diurnal activity, and assess seasonal variation over the study period (~6 months). We also aimed to evaluate an often-neglected aspect of energetic requirements by quantifying cumulative distances moved by individual animals over extended periods of time.

## Methods

### Study Area

**Figure 1:**
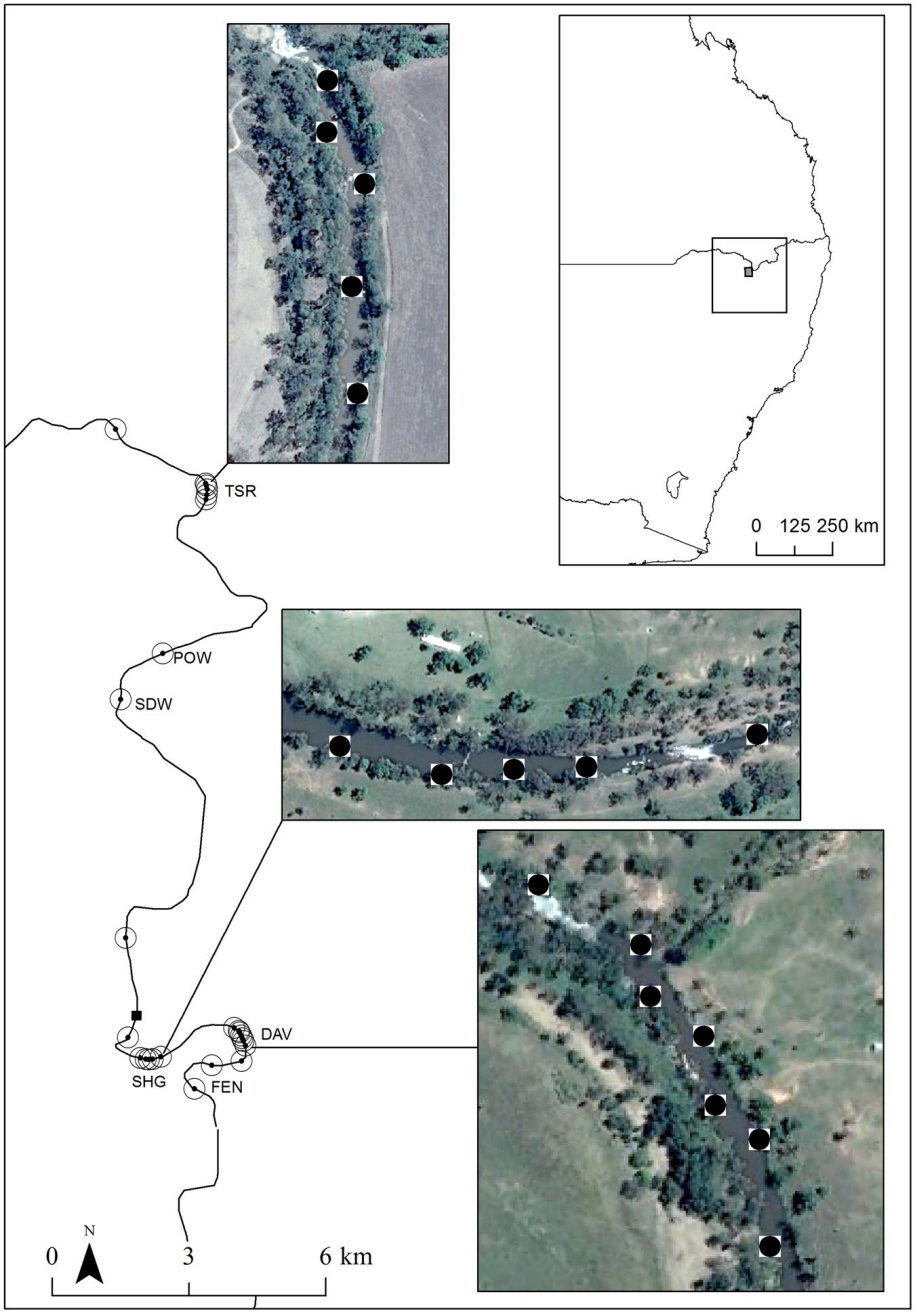
Location of the Severn River (NSW), Australia, where nine platypuses were trapped at six pools (TSR, POW, SDW, SHG, DAV, FEN) and implanted with acoustic transmitters, detectable by 25 acoustic receivers (black dot and circle), deployed along the river to detect fine scale movements in three pools (inset photographs). Black square indicated the location of the Ashford River gauge (416006) on the Severn River (NSW), Australia. Images show detailed locations of acoustic receivers (black circle) in pools selected for fine scale tracking. Figure was generated using ArcGIS 10.3^79^ and images from Google Earth (© 2018 Google, Image © 2018 CNES/Airbus, Image © 2018 DigitalGlobe).

We studied platypus movements in pools along about 25 km of the Severn River, a regulated river located in northern NSW, Australia (Fig. 1). The Severn River (235 km long) flows northwest from its headwaters in the Great Dividing Range in northern New South Wales. Most flows are regulated by Pindari Dam (312,000 ML), except for flows from downstream Frazers Creek, before the river joins (85 km downstream of the dam) the Macintyre River, part of the Murray-Darling Basin. Grazing is the primary land use in the catchment area, with about 60% of land cleared of native vegetation, with some areas of remnant woodlands of the Nandewar Bioregion (Hunter *et al*. 1999), surrounding the Severn River. Typical riparian tress include: Blakely’s red gum (*Eucalyptus blakelyi*), river oak (*Casuarina cunninghamiana)*, rough-barked Apple (*Angophora floribunda*), and yellow Box (*Eucalyptus melliodora*)^30^.

### Platypus trapping and implants

We trapped platypuses in pools using unweighted mesh nets^31^, set an hour before dark, until about 01:00 (Australian Eastern Daylight Saving Time) hours. Pools were similar along the river, with trapping carried out in those most accessible. Width of pools ranged between 30 m and 50 m and lengths from 300–600 m. For nets to be effective and safe for platypuses, pools had a minimum water depth of 2 m with some sections up to about 5 m. Captured animals were removed from the water and held in pillow cases prior to anaesthetic induction in an induction chamber, using isoflurane (Pharmachem) (5%) in oxygen (3 L/min), (Darvall DVM ISO)^32–34^. Platypuses were then maintained under anaesthesia, via a T-piece and face mask, supplying isoflurane (1–2.5%) in oxygen (1.0 L/min)^35^. Blood oxygen saturation, heart rate, and body temperature were continuously monitored (Darvall H100N).

Between the 22^nd^ Jan and 12^th^ of Feb 2016, we trapped 14 platypuses: five females and nine males. Females could not be aged beyond 9 months but one male juvenile (<1 year) and one sub-adult (~1–2 years) were identified by their spur morphology^36^. The sub-adult male would likely have become sexually mature within the study period^37^. We implanted Vemco miniature acoustic tracking transmitters (model V7–4L (Vemco Limited, Nova Scotia, Canada) into the peritoneal cavity of nine platypuses (three females, six males (one juvenile and one sub-adult), Table 1), following reported implantation methods used in platypuses, with good healing and without infection^29,38,39^. Although less reliable than injecting synthetic oxytocin to increase milk let down^40^, we tried to avoid implanting lactating females by physically examining them for signs of lactation, with milk being detected in a single female, which was not implanted with an acoustic tag. Although specific guidelines do not exist for implanted devices^41–43^, miniature acoustic tracking devices were well within available recommended guidelines for external devices (5–10% of individual body mass *American Society of Mammalogists*^43^). Our transmitters were 22.5 mm length, 7 mm diameter, weight in air 1.8 g (weight in water 1.0 g), about 0.15% of average adult females’ body weight (1.22 kg ± 0.14 SD) and 0.11% of adult males’ body weight (1.67 kg ± 0.17 SD), (Table 1). Tag expected battery life was 197 days (~6.5 months; March-August), which would align with possible long-distance movements by emerging juveniles^4^. All implants were coated with a physiologically compatible wax, to minimise tissue reaction (elvax mixture: 80% paraffin wax, 20% elvax by weight), after heating to about 100 °C and placed in plastic bags with chlorhexidine solution, when cooled.

**Table 1:** The nine platypuses implanted with acoustic tags, their weight, tagging date, first and last day of detection, number of detection and number of days detected of the nine platypuses implanted with acoustic tags.

ID	Sex	Weight (kg)	Tagged	First detection	Last detection	N detections	N days
SHG1	Male	1.83	12/2/2016	22/2/2016	31/8/2016	9170	188
SHG2	Male/J	1.50	12/2/2016	14/2/2016	5/8/2016	147	173
FEN	Male/SA	1.43	10/2/2016	14/2/2016	29/8/2016	596	197
SDW*	Female	1.34	27/1/2016	1/2/2016	16/8/2016	2120	197
POW*	Male	1.77	24/1/2016	31/1/2016	30/7/2016	48	181
TSR	Male	1.80	23/1/2016	1/2/2016	12/8/2016	5491	193
DAV1	Female	1.24	22/1/2016	NA	NA	0	0
DAV2	Female	1.07	22/1/2016	4/2/2016	11/8/2016	1860	189
DAV3	Male	1.66	22/1/2016	29/1/2016	11/8/2016	10,714	195

Platypuses were placed in dorsal recumbency and a small area (5 mm × 15 mm longitudinally) of fur removed over the ventral midline, about half way between the *xiphisternum* and *pubis*, using clippers. The surgical site was prepared using three alternating applications of 70% methanol and diluted chlorhexidine solution (0.1% w/v aqueous solution). We followed full sterile surgical procedures including the use of disposable gloves and scalpel blades. A sterile paper drape with a 5 × 15 mm central hole cut was used and secured at its edges to the fur, with small drops of instant adhesive (Selleys Quick Fix). A 10 mm skin incision, using a size 15 scalpel blade, was made through the skin, subcutaneous tissue and superficial *cutaneous trunci* muscle, down to the *linea alba*, through which an 8 mm incision was made into the *peritoneum*. The transmitter was flushed with sterile saline solution and inserted into the *peritoneal* cavity. The *linea alba* incision was closed with 3–4 single interrupted sutures, using 3–0 monofilament absorbable suture material (Ethicon Z442H). A few drops of bupivacaine hydrochloride (Pfizer Bupivacaine, 0.1 ml), a local anaesthetic, were then applied to the wound before the skin was closed with 1–2 cruciate sutures, using 3–0 monofilament absorbable suture material, followed by application of cyanoacrylate topical tissue adhesive to seal the incision (3 M Vetbond 1469 C).

### Platypus tracking and statistical inference

Twenty-five receivers (VR2W-069k) were placed along the Severn River, over 27 km (Fig. 1), between the 1^st^ of March and 31^st^ of August 2016. Field testing of acoustic tags with wax coating indicated detection ranges of 25 m in a deep (5 m) pool. Placement of receivers was designed to meet two objectives. First, identification of long distance movements along the river required receivers about 2–3 km apart, except where access restrictions required greater distances (Fig. 1). Second, we aimed to track fine scale movement within pools, with receivers in three pools, spaced 50–100 m apart (Fig. 1). We placed seven, five and five receivers in pools DAV, SHG and TSR respectively (Fig. 1). Detections represented the number of times a receiver ‘detected’ an individual platypus. Distance between detection was calculated as distance along the river, between receivers (zero was assigned for detections by the same receivers). We calculated distances at different temporal intervals: daily, weekly, and monthly. We calculated and analysed several metrics relating to movement and habitat use: 1) maximum distances between detections, calculated at daily (24 h cycle from 13:00), weekly and monthly (30 days) spans; 2) cumulative movement, the sum distance of all sequential receiver records, calculated at daily, weekly and monthly spans and; 3) diurnal and nocturnal activity, calculated as the frequency of records in each hour of day (platypuses were undetected while out of water). To test for spatio-temporal autocorrelation among sequential maximum distances, daily detections and cumulative daily movements, we used the non-parametric autocorrelation function (ACF) indicating significance if greater than two standard errors from zero^44^. Animal movements are a non-independent behaviour^45^, resulting in strongly autocorrelated data, particularly when using frequent tracking data as in our study. We avoided analysis that eliminated autocorrelation to satisfy non-autocorrelative assumptions, as this would have reduced the relevance of analysis and drawing meaningful insights into movement behaviour^45,46^.

Impacts of implants may include short-term effects such discomfort and pain or medium-term effects such as inflammatory processes that can be monitored using physiological (e.g., body temperature) or behavioural (e.g., accelerometers) transmitters^47^; these were not used as they required larger implants, beyond our preference. As we only included data from the 1^st^ of March, over two weeks post-surgery and release of platypuses (Table 1), possible short-term impacts of the surgery on behaviour would likely be limited. Comparative studies of both externally glued and implanted tags could provide the means to examine short and medium-term effects while long-term survival would be challenging given inability to accurately age platypuses and low recapture rates in most platypus populations.

We investigated whether circadian activity associated with time of day and season (i.e., months, March-August) were related to total number of hourly detections as the response variable. We used a Generalized Additive Mixed Effects Models, incorporating hour (24 hr) as a continuous explanatory variable, with an interaction term of month as a fixed effect explanatory factor (i.e., categorical) and individual platypus as a random effect factor (i.e., individual platypuses are a random sample from a larger population with an aim to make a statement regarding the larger population). We also incorporated average monthly river height (Ashford River Gauge 416006^48^) and average rainfall (Beaumont 054043^49^) (Appendix 1) as continuous explanatory variables to control for potential environmental confounding variables (i.e. affecting interpretation of relationships) on detection frequency^50,51^. Generalized Additive Models (GAMs and GAMMs) are a compromise between a linear model and a smoothing function, making them a flexible tool with few statistical assumptions^52^. Predictors depend linearly on unknown smooth functions of some of the covariates^53^, with the degree of smoothing selected by minimum GCV/UBRE scores, controlling for over-fitting with a gamma multiplier of 1.4^54^. To avoid overfitting and ease of interpretation, we limited the number of knots (i.e., polynomial level) in the GAMs to three^55^. To account for imbalanced samples sizes among individuals, we used the Restricted Maximum Likelihood Estimation (REML)^56^ and included individual platypuses as either a fixed or random variable in all our models. We used a GAMM, with a negative binomial link function, using the ‘gamm’ function in the ‘mgcv’ package^57^ in the R environment^58^. The negative binomial link function (i.e., the relationship between the linear predictor and the mean of the distribution function^59^) was used, given that the dependant data were discrete occurrences among discrete trials (hours of day). Further, we used the likelihood ratio test to compare Poisson and Negative Binomial distributions, using a chi-squared distribution which indicated a significantly larger log-likelihood when using a Negative Binomial distribution (χ^2^ = 13811, df = 1, *P* < 0.001).

Additionally, we investigated whether daily maximum and daily cumulative distances varied over months (i.e., March – August). To do this, we used a GAM and the ‘gam’ function in the ‘mgcv’ package^57^ in the R environment^58^ to test possible association with month, water level, and rainfall as continuous explanatory variables. We also included total number of daily records, given the likely dependence of daily cumulative distances. The Variance Inflation Factor was examined for explanatory variables, but was well below (<2) the threshold of 10, indicating low collinearity^60^. To account for variation among individual platypuses, we included interaction terms between both month and water level, with individual platypus (factor). We used the likelihood ratio test to compare the log transformed (ln(x + 1)) and untransformed daily maximum and daily cumulative distances to identify the best fit with Gaussian distribution, with GAM. Log-transformed models had a significantly higher log-likelihood model performance (χ^2^ = 7812.5, df = 0.18, *P* < 0.001 and χ^2^ = 1425.8, df = 1.23, *P* < 0.001), indicating a better fit with a log-transformed data.

Trapping and handling of platypuses followed guidelines and approval of the NSW Office of Environment and Heritage (SL101655), NSW Department of Primary Industries (P15/0096–1.0 & OUT15/26392), and UNSW’s Animal Care and Ethics Committee (16/14 A).

## Results

Nine platypuses (three adult females, four adult males, one sub-adult male, and one juvenile male) were tagged with acoustic transmitters but one female remained undetected throughout the study period (Table 1). Given the female was not detected immediately after release by the receiver next to the release site, this indicated likelihood of transmission failure, rather than an adverse event. All receivers detected platypuses, which indicated functionality. The number of detections varied among the other eight individuals, ranging from 48 to 10,714 (male average 4,361 ± 4,802 sd, female average 1,990 ± 184 sd, one male sub-adult 596, and one male juvenile 147) over the six-month period.

**Figure 2:**
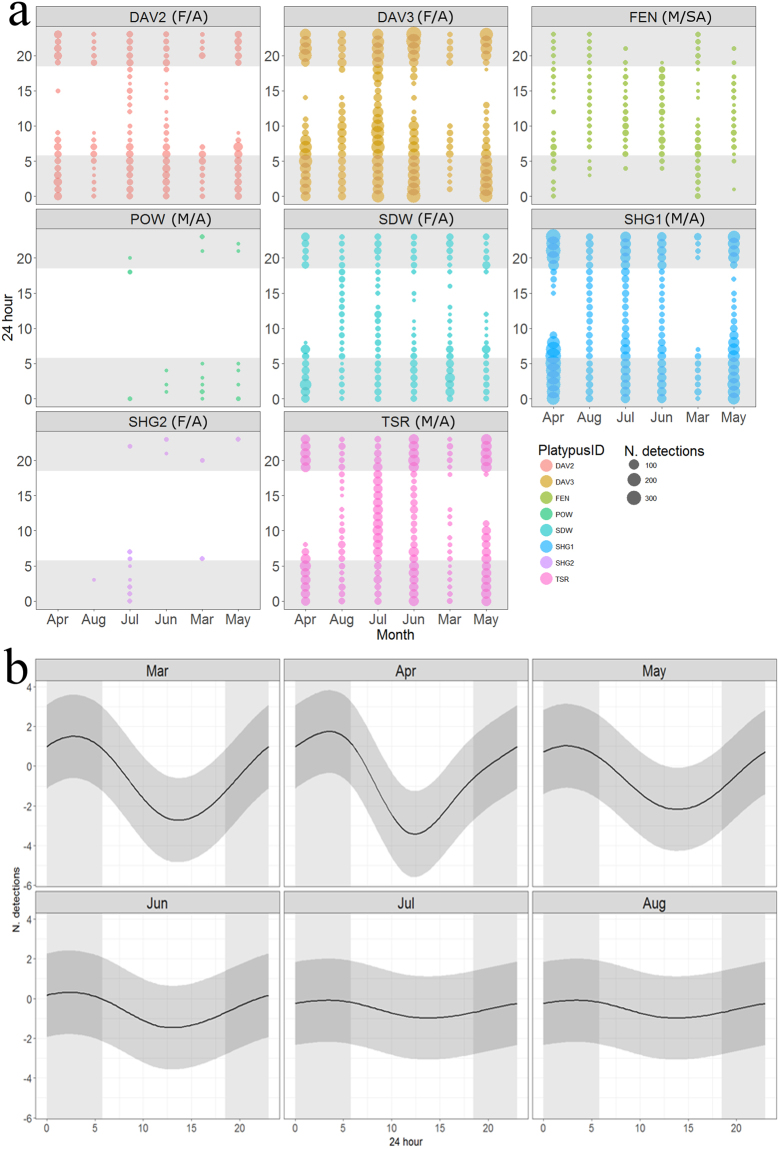
(**a**) Observed number of detections (size of circle) at each hour (24 hr), March-August, panelled and coloured by individual platypuses (see Table 1 for IDs) and (**b**) estimates of circadian activity (number of detections) at each hour during each month, based on the Generalized Additive Mixed-Effect Model, which indicated significant associations between activity and time of day, March-June but not July-August (see Appendix 2 for model outputs). Average sunrise\sunset times March to August (5:47/18:29) indicated by grey background shading.

There was a clear pattern to circadian activity (i.e., number of detections), indicated by a significant association between time of day, starting from 20:00, about two hours after sunset (average time of sunset 17:51) and ending at 7:00, an hour after sunrise (average sunrise: 06:17), (Fig. 2 and Appendix 2). Significant association between activity and time of day was present between March and June but not between July and August due to an increased frequency of diurnal activity in winter (Fig. 2 and Appendix 2).

**Figure 3:**
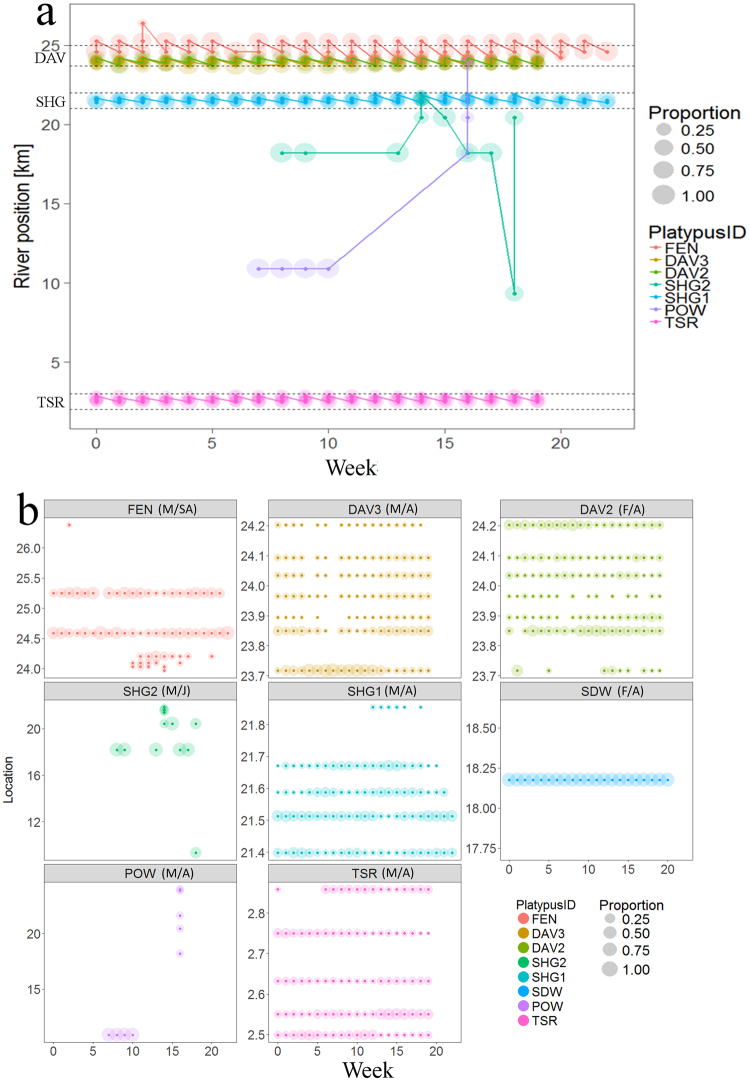
Proportion (circle size) of weekly (1–23, Mar-Sept) records in each of the receivers along the 0–27 km of the Severn River (i.e., river position of receiver) f for the eight platypuses (colour): (**a**) across the entire river section with pools, where pools with multiple receivers marked with a dashed line and labelled accordingly and (**b**) repeated for each individual platypus (see Fig. 1).

There was considerable variation in movements of individuals, with most adult platypuses exhibiting high fidelity to pools where they were captured, while juvenile and sub-adult platypuses showed comparatively extensive movements (Fig. 3). For example, adult male SHG1 was not recorded outside pool SHG, while juvenile male SHG2 made two forays 2.25 km upstream followed by a downstream movement of 11.11 km (Fig. 3). Adult male POW was recorded in pool POW for five months but then moved upstream 13 km, then downstream 5.8 km. Adult male TSR was not recorded outside pool TSR. Both adult female DAV2 and adult male DAV3 were not recorded outside pool DAV and did not show any variation in spatial use of the pool over time. Sub-adult male FEN was observed regularly in both pools FEN and DAV (2 km downstream), (Fig. 3). Sequential daily maximum movements were autocorrelated among individuals, although significant positive autocorrelation was only detected in three male platypuses, up to lag of 20 days (DAV3, SHG1 and TSR).

**Figure 4:**
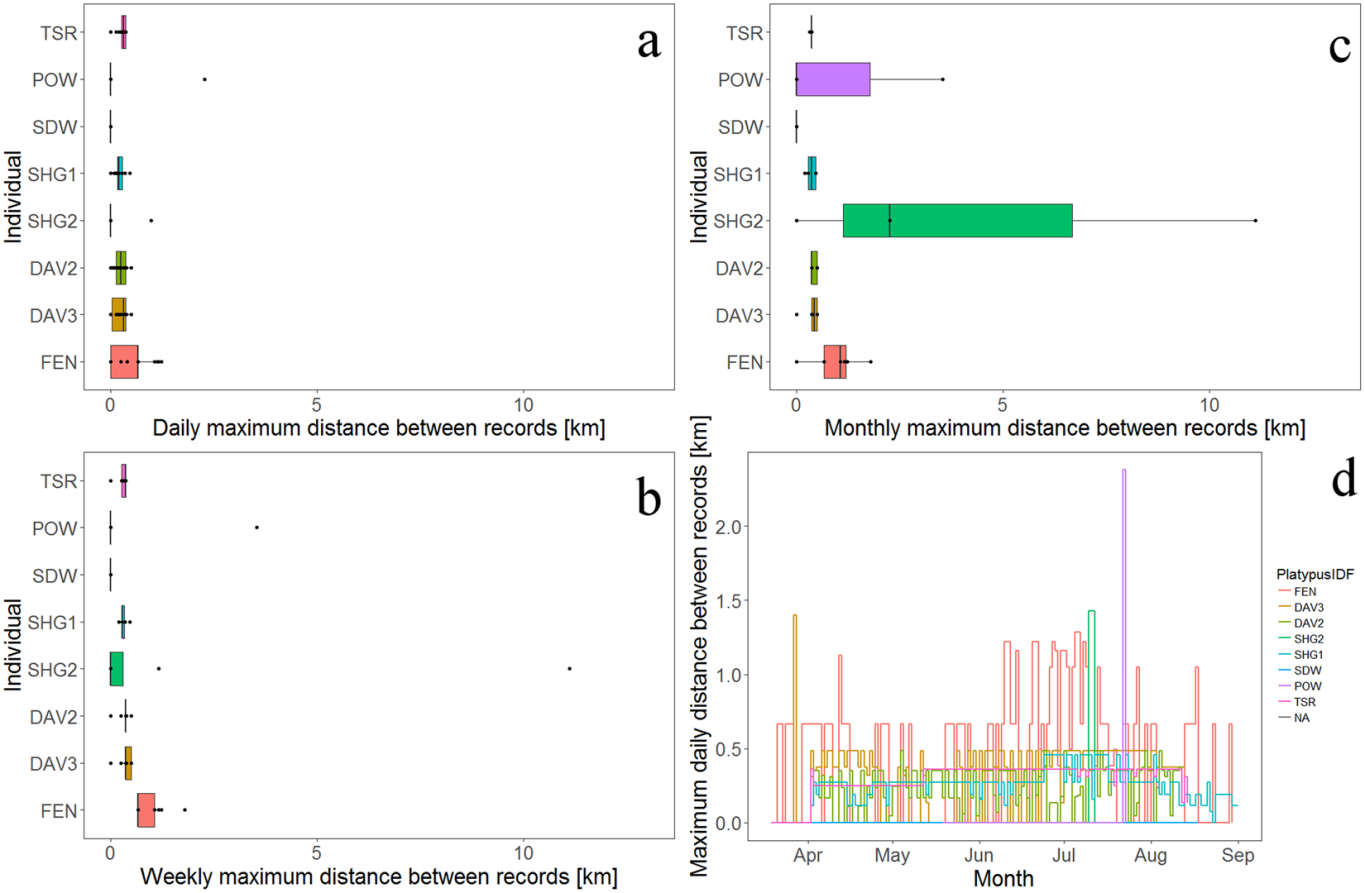
Box plots of maximum distances between detections [km] for the eight platypuses estimated for (**a**) daily, (**c**) weekly, and (**c**) monthly periods, and (**d**) time series line plot of daily maximum distances for each platypus (April-August) (see Table 1 for IDs matching the individuals).

There were variable relationships among months, water levels and maximum recorded daily movements, identified by the Generalized Additive Model. Total number of daily occurrence records was associated with maximum daily distances, with an asymptotic relationship, levelling towards 100 daily records (F = 68.6, P < 0.001, Appendix 3). Maximum daily distances were also positively associated with both month (F = 5.71, P = 0.002) and water level (F = 11.27, P < 0.001, Fig. 4 and Appendix 3). Maximum daily distances between detections were relatively similar among adult platypuses in pools with multiple receivers (n = 4), with a mean of 239 m ± 34 sd (range: 360–485 m), (Fig. 4 and Table 2). Notably, juvenile and sub-adult males moved up to a maximum daily distance of 972 m (SHG2) and 1.2 km (FEN) but with lower averages and greater variability than adult males and females: average of 0.097 km ± 0.307 sd (SHG2) and 0.421 km ± 0.396 sd (FEN), respectively (Table 2). Adult male POW travelled a maximum daily distance of 2.26 km (Fig. 4 and Table 2). Mean maximum weekly distances between detections of adult platypuses in pools were greater compared to juveniles and sub-adults respectively 329 m (±3 sd), 1.53 km (±3.89 sd) and sub-adults 0.89 km(±0.30 sd) males (Fig. 4 and Table 2); the latter travelled a maximum distance of 11.1 km in three days (Fig. 2a, Fig. 4, Table 2). Mean maximum monthly distances between detections increased slightly over daily and weekly time frames, for all adult platypuses (mean 368 m ± 3 sd) but considerably for the juvenile (SHG2: 4.45 km ± 5.87 sd) and sub-adult FEN (0.94 km ± 0.56 sd) males (Fig. 4 and Table 2).

**Table 2:** The mean (±sd)/range/sample size of maximum distances [km] between detections at daily, weekly, and monthly scales for the eight platypuses detected with acoustic tags.

ID	Sex	Day	Week	Month
SHG1	Male	0.21 ± 0.08/(0–0.46)/155	0.29 ± 0.08/(0.19–0.46)/23	0.35 ± 0.12/(0.19–0.46)/6
SHG2	Male/J	0.10 ± 0.31/(0–0.97)/11	1.53 ± 3.89/(0–11.11)/8	4.45 ± 5.87/(0–11.11)/4
FEN	Male/SA	0.42 ± 0.40/(0–1.22)/134	0.89 ± 0.30/(0.67–1.79)/23	0.94 ± 0.56/(0.67–1.79)/6
SDW*	Female	0 ± 0/(0–0)/ 143	0 ± 0/(0–0)/ 22	0 ± 0/(0-0)/ 6
POW*	Male	0.17 ± 0.63/(0-2.26)/13	0.51 ± 1.34/(0–3.54)/5	1.18 ± 2.04/(0–3.54)/3
TSR	Male	0.29 ± 0.07/(0–0.36)/136	0.31 ± 0.09/(0–0.36)/20	0.35 ± 0.02/(0–0.36)/6
DAV2	Female	0.22 ± 0.13/(0–0.49)/133	0.36 ± 0.10/(0–0.49)/21	0.41 ± 0.07/(0–0.49)/6
DAV3	Male	0.24 ± 0.16/(0–0.49)/138	0.36 ± 0.14/(0–0.49)/20	0.36 ± 0.19/(0–0.49)/6

^*^One receiver in pool.

**Figure 5:**
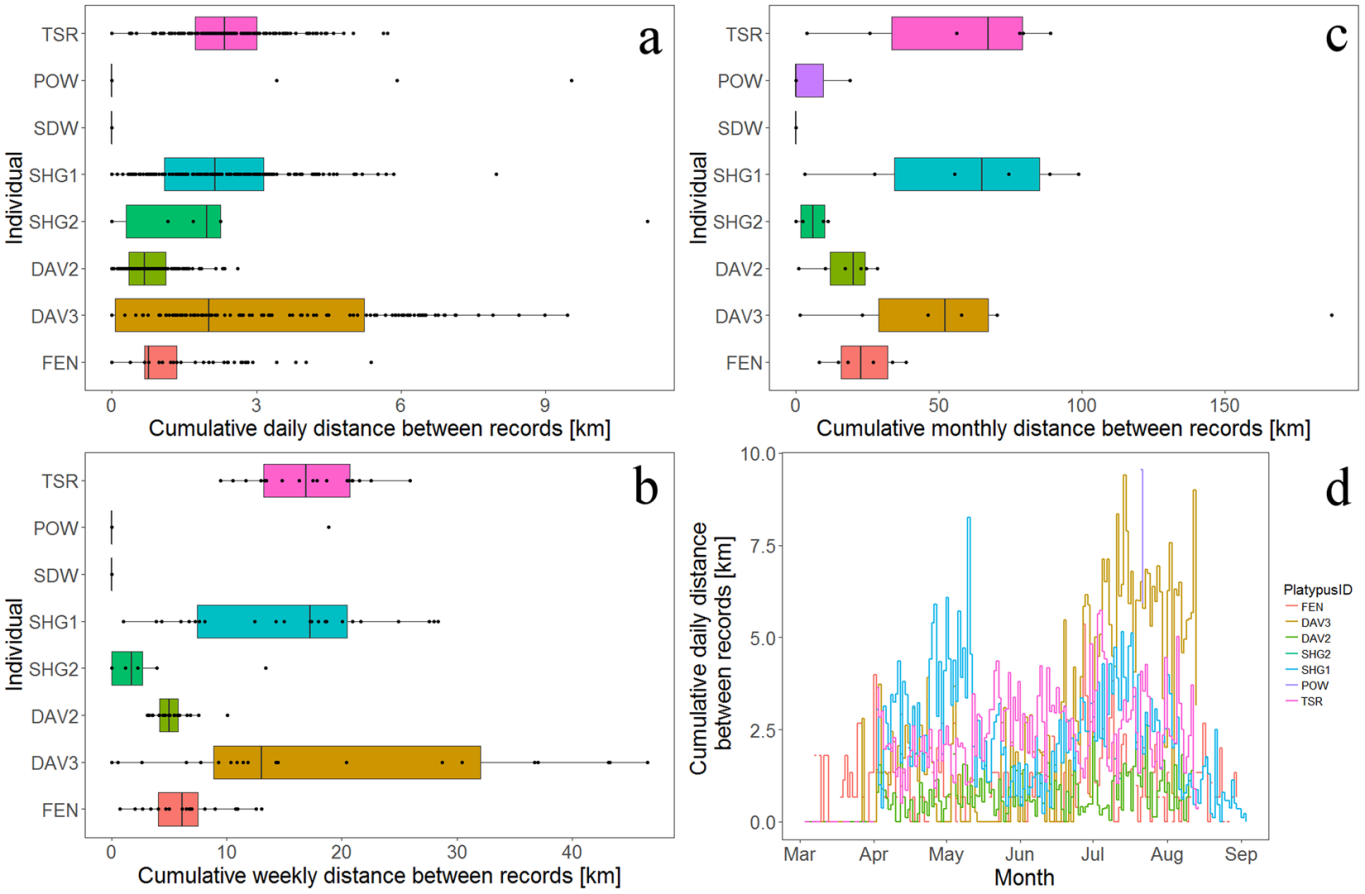
Box plots of cumulative daily movements distances [km] between detections for the eight platypuses estimated for (**a**) daily, (**b**) weekly, and (**c**) monthly periods and (**d**) time series line plot of daily cumulative distances for individual platypuses (see Table 1 for IDs matching the individuals).

There were differences among the different demographic groups and sex at different time scales. Mean cumulative movement between consecutive records of the adult female DAV2 was considerably lower compared with other adult males, ranging between 27% and 34% of mean male movements (Table 3). Daily Mean cumulative movement of female DAV2 was 783 m (±555 sd), compared to adult male distances 2.3 km–2.8 km (Fig. 5 and Table 3). Similarly, maximum cumulative daily movement of the adult female was 2.6 km, compared to adult males 5.7km–9.4 km (mean of 8.23 km ± 2.27 sd, Fig. 5 and Table 3). Juvenile and sub-adult males had a mean cumulative daily movement of 2.30 km (±3.25 sd) and 1.10 km (±0.97 sd), respectively while their maximum cumulative daily movement was 11.1 km and 5.4 km, respectively. At the scale of weeks, mean cumulative movements of the adult female DAV2 were 5.2 km (±1.67 sd, maximum 10.1 km) and mean cumulative weekly movements of the three adult males ranged between 15.6 km (±8.56 sd) and 19.3 km (±15.32 sd, maximum 26 km to 46.6 km) for the adult males. Juvenile and sub-adult males had a mean cumulative weekly movement of 2.8 km (±4.46 sd) and 6.1 km (±3.43 sd) and respective maxima of 13.4 km and 13.1 km. Monthly mean cumulative movement of the adult female DAV2 was 17.4 km ± 10.35 sd (maximum 28.5 km), while those of the three adult males ranged between 55.5 km (±34.04 sd) and 64.3 km (±65.09 sd, maximum 89.2 km–187.1 km).

**Table 3:** The mean (±sd)/range/sample size of cumulative distances [km] between detections at daily, weekly, and monthly scales for the eight platypuses detected with acoustic tags.

ID	Sex	Day	Week	Month
SHG1	Male	2.32 ± 1.47/(0–8.24)/155	15.56 ± 8.55/(1.03–29.54)/23	59.64 ± 38.09/(3.04–101.15)/6
SHG2	Male/J	2.30 ± 3.25/(0–11.11)/11	2.87 ± 4.46/(0–13.36)/8	5.74 ± 5.44/(0–11.11)/4
FEN	Male/SA	1.10 ± 0.97/(0–5.36)/134	6.07 ± 3.43/(0.67–13.05)/23	23.26 ± 11.70/(7.99–38.40)/6
SDW^*^	Female	0/(0)/143	0/(0)/22	0/(0)/6
POW^*^	Male	1.45 ± 3.03/(0–9.54)/13	3.78 ± 8.44/(0–18.87)/5	6.29 ± 10.90/(0–18.87)/3
TSR	Male	2.43 ± 1.08/(0–5.73)/136	16.64 ± 4.77/(9.5–25.97)/20	55.46 ± 34.04/(9.5–89.18)/6
DAV2	Female	0.78 ± 0.56/(0–2.61)/133	5.21 ± 1.66/(3.06–10.09)/21	17.35 ± 10.35/(3.06–28.52)/6
DAV3	Male	2.80 ± 2.56/(0–9.41)/138	19.28 ± 15.32/(0–46.60)/20	64.27 ± 65.09/(1.41–187.14)/6

^*^One receiver in pool.

Sequential daily cumulative movements were positively autocorrelated for male DAV3 up to a lag of 30 days followed by negative autocorrelation from lag 50 days because of increased daily cumulative movements from mid-June. There were also positive autocorrelations in the daily cumulative movements of male SHG1, with lag up to 16 days. In addition, short term positive autocorrelation was observed for FEN (lag 2 days) and TSR (lag 3 days). There were variable relationships between cumulative daily movement in relation to months and water levels, indicated by the Generalized Additive Model. Total number of daily occurrence records was highly associated with cumulative daily movement, with an asymptotic relationship above 150 daily records (F = 399.4, P < 0.001, Appendix 4). Similarly, water level at Ashford Gauge (Fig. 1), ranging between 0.23 m and 2.56 m (Appendix 1), was positively associated with cumulative daily movement up to about 1.0 m (F = 10.9, P < 0.001, Appendix 4). Month had a marginal positive association with cumulative daily movement, increasing towards June, following a slight decrease in cumulative daily movement (F = 2.9, P = 0.088, Appendix 4). Rainfall was not associated with cumulative daily movements (F = 0.01, P = 0.914, Appendix 4).

## Discussion

Implanted acoustic transmitters in platypuses and the linear array of acoustic receivers effectively tracked eight platypus movements over six months, providing both fine scale and broad movement data. We recorded a wide range of movement patterns, with movements of four of the five adult platypuses constrained to relatively local scales, with monthly ranges of about 400 m (Table 2). Contrastingly, one adult (POW), sub-adult and juvenile male platypuses moved greater distances compared to other adult platypuses across different temporal periods, with average monthly ranges extending to 1.2 km, 950 m and 4.5 km, respectively (Table 2 and Fig. 2).

Our continuous data substantially improved current investigations of platypus movements, providing continuous tracking over a relatively extended period. It also contributed to current understanding that platypus movements and activities vary among individuals^11,16,17,29,61^. Over six months (March to August), outside the species’ breeding season^12,62^, our detailed data showed that most adult platypuses exhibited high fidelity to pools, foraging areas, in terms of maximum daily distance travelled, increasing over time (Table 2, Fig. 4 and Appendix 3). There was also strong evidence that activity patterns changed from predominantly nocturnal to inclusion of some diurnal foraging in the winter months of June to August (Fig. 3). Such shifts have previously been observed in platypuses foraging in several locations, attributed to resource variability when energy requirements increase with seasonal regulation of body temperature, as well as territoriality during the breeding season (around September)^13,17,63–65^. Uncertainty still exists over quantity, composition and variation of platypus diet and differentiation between summer and winter periods^66,67^. Emerging stable isotope analysis in platypuses^68^ and DNA-based identification of macroinvertebrate prey items^69^ will undoubtedly improve our understanding of seasonal changes in dietary composition. Notable fine scale (<500 m) variation in activity of some individuals (Fig. 2b) may indicate focal foraging areas reflected in food availability, although spatial exclusion or separation by other male platypuses may also be at play^16^.

We were also able to contribute more to understanding dispersal and establishment of juveniles, with evidence that sub-adult (FEN) and juvenile (SHG2) males regularly travelled greater distances than adults, visiting multiple pools over the study period (Fig. 2a). This supports mark-recapture studies of juvenile male platypuses^12,70^, where one individual travelled 44 km, over 30 weeks^70^. Juvenile male DAV2 made several forays from the site of initial tagging, possibly attempting to establish home range (Fig. 2) or perhaps displaced by other adult territorial males, forcing separation for competition to females during the breeding season^4,36,37^. Possibly related, adult male POW made long-distance forays over several weeks (Table 2, Figs 2 and 4), travelling a maximum distance of 13 km upstream and a total of about 19 km, with 11.1 km travelled in three days. Accurate estimates of movements and home ranges were largely dependent on placement and number of receivers, representing lower bounds of distances travelled.

Use of metabolic rates to determine carrying capacity of rivers for platypus populations may also be affected by the considerable cumulative movements of individuals that we detected (Table 3 and Fig. 5). Daily field metabolic demand of platypuses has been estimated in two studies, using time-energy budgets based on measurements of oxygen consumption in the laboratory on platypuses from the upper Shoalhaven River in NSW^71^, and metabolic rates determined in a laboratory swim tank, supported by foraging behaviour using data-loggers in a sub-alpine lake in Tasmania^18,72^. Metabolic demands estimates differed by more than a factor of two, between studies. Using a doubly labelled water technique in the same sub-alpine lake in Tasmania calculated daily metabolic demand around 30% higher^73^. Recently, benthic macroinvertebrate productivity, a predominant food source for platypuses^68,74^, was used along with the two published values of daily field metabolic demand for the platypus^18,72^ to calculate potential carrying carry capacities for a 1.5 km reach in the upper Shoalhaven River (NSW), reporting lower capacities during a dry low flow year (13–27; 2009) than in a year of medium stream flows (22–45; 2011)^66^. However, we identified considerable variation in movements among individual platypuses (Table 3, Fig. 5), as elsewhere reported. In the southern distribution of the platypus, daily foraging areas varied between 3 and 58 ha in a Tasmanian lake^61^ and between 0.4 to 6.2 km in a Victorian river system^15^. Although the potential platypus carrying capacity estimated using benthic macroinvertebrate productivity and metabolic demand^66^ fell within the range of mark-recapture population estimates for the upper Shoalhaven River^12^, we caution against reliance on such estimates alone for conservation or management^75^.

Implantation of acoustic tags was a powerful method for investigating this elusive and poorly studied species, offering new avenues for examining behaviour relating to mating and nesting, energetic expenditure, and competition through spatial and temporal displacement. Given emerging understanding of dependence between flows and platypus breeding^7,12,76^ and the positive associations between cumulative movements and river flows, continuous tracking using acoustic implants could valuably assess impacts of river regulation (building dams and diverting water) which may reduce macroinvertebrate prey species availability, threaten juveniles confined to nesting burrows and increase the energy demands for platypuses foraging in high flows, particularly lactating females and newly emerged juveniles. Use of receivers within intersecting detection range could triangulate exact localities of individuals for fine scale behaviour^77^. Also, investigations of juvenile dispersal^12,70^ over long periods of time could be achieved by reducing signal frequency and extending battery life (e.g. 12 months). Emerging technologies are rapidly progressing understanding of aquatic animal behaviour, supporting conservation management^78^. Implantable acoustic tags provide a powerful new technique for studying this unique aquatic mammal but are still constrained by battery life.

## Electronic supplementary material

Supplementary information
