# Supplementary material for: Use of implanted acoustic tags to assess platypus movement behaviour across spatial and temporal scales
Source: Sci Rep. 2018 Mar 23;8:5117. doi: 10.1038/s41598-018-23461-9 (PMC5865170; doi:10.1038/s41598-018-23461-9)

**Use of implanted acoustic tags to assess platypus movement behaviour across various spatial and temporal scales**

Gilad Bino^*1^, Richard T. Kingsford^1^, Tom Grant^1^, Matthew D. Taylor^2^, Larry Vogelnest^3^

# Appendix 1: water level

Daily water level (m) measured at Ashford River Gauge (416006) on the Severn River (grey shaded) and daily rainfall [mm] measured at Ashford Town (Beaumont - 054043), Jan-Sept 2016. See Figure 1 for location.


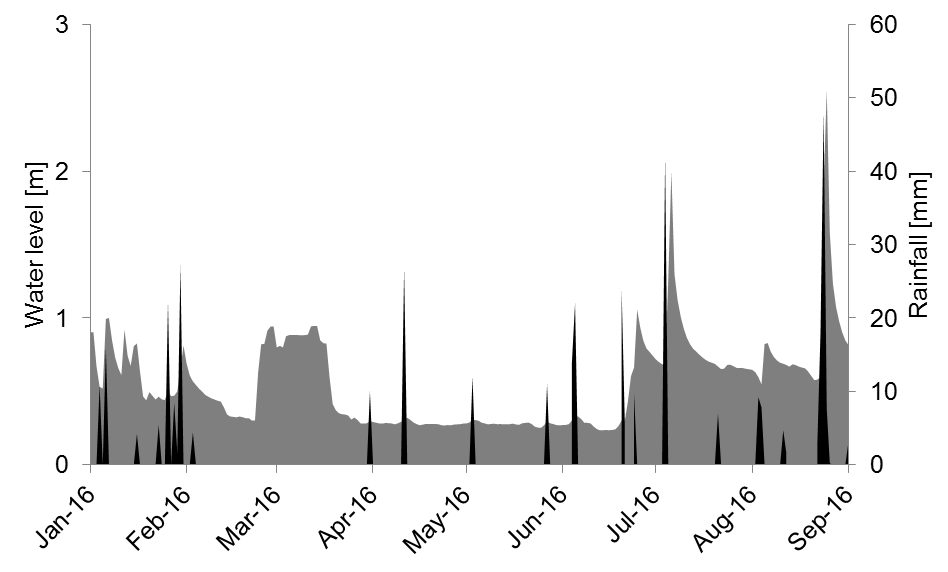


# Appendix 2: activity

Results of Generalized Additive model of activity (proportion of total number of hourly records, calculated for each individual platypus separately) in response to time of day, month, water level, and rainfall.

| Parametric coefficients: | Estimate | se | t | P |
| --- | --- | --- | --- | --- |
| Intercept | 1.479 | 0.7578 | 1.953 | 0.051 |

| Smooth terms: | edf | Ref.df | F | P |
| --- | --- | --- | --- | --- |
| Water level | 1.000 | 1.000 | 8.95 | 0.003 |
| Rainfall | 3.312 | 3.312 | 57.40 | <0.001 |
| Time | 2.391 | 3.000 | 9.71 | 0.000 |
| Time*March | 2.693 | 3.000 | 32.90 | <0.001 |
| Time*April | 2.883 | 3.000 | 51.56 | <0.001 |
| Time*May | 2.567 | 3.000 | 20.77 | 0.000 |
| Time*June | 1.950 | 3.000 | 3.88 | 0.001 |
| Time*July | 0.001 | 3.000 | 0.00 | 0.838 |
| Time*August | 0.001 | 3.000 | 0.00 | 0.848 |

# Appendix 3: maximum daily distance

Model coefficients (table) and predicted responses (plots) of Generalized Additive Model of the association between maximum daily distance in response to month, water level, rainfall, and total counts with an interaction term between individual platypuses and month and water level.

| Parametric coefficients: | Estimate | S.E. | t | P |
| --- | --- | --- | --- | --- |
| Intercept | 3.9 | 0.082 | 47.46 | <0.001 |

| Smooth terms: | Edf | Df | F | P |
| --- | --- | --- | --- | --- |
| Water level [m] | 1.00 | 1.00 | 11.27 | <0.001 |
| Rainfall [mm] | 1.00 | 1.00 | 1.03 | 0.31 |
| Counts | 1.95 | 2.00 | 68.58 | <0.001 |
| Month | 1.88 | 1.98 | 5.71 | <0.001 |
| Wlevel:DAV2 | 1.00 | 1.00 | 12.70 | <0.001 |
| Wlevel:DAV3 | 1.55 | 1.79 | 4.11 | 0.01 |
| Wlevel:FEN | 1.93 | 2.00 | 10.40 | <0.001 |
| Wlevel:POW | 0.00 | 0.00 | 1.10 | 1.00 |
| Wlevel:SDW | 1.00 | 1.00 | 10.35 | <0.001 |
| Wlevel:SHG1 | 1.00 | 1.00 | 11.07 | <0.001 |
| Wlevel:SHG2 | 1.00 | 1.00 | 2.49 | 0.11 |
| Wlevel:TSR | 1.00 | 1.00 | 10.35 | <0.001 |
| Month:DAV2 | 0.00 | 0.00 | 0.38 | 0.98 |
| Month:DAV3 | 1.87 | 1.98 | 4.28 | 0.01 |
| Month:FEN | 1.00 | 1.00 | 14.60 | <0.001 |
| Month:POW | 1.00 | 1.00 | 3.61 | 0.06 |
| Month:SDW | 1.00 | 1.00 | 0.92 | 0.34 |
| Month:SHG1 | 1.00 | 1.00 | 0.21 | 0.65 |
| Month:SHG2 | 1.84 | 1.97 | 6.11 | <0.001 |
| Month:TSR | 1.00 | 1.00 | 1.37 | 0.24 |

R-sq.(adj) = 0.205 Deviance explained = 22.6%, -ML = 1972.3 Scale est. = 5.6405 n = 864


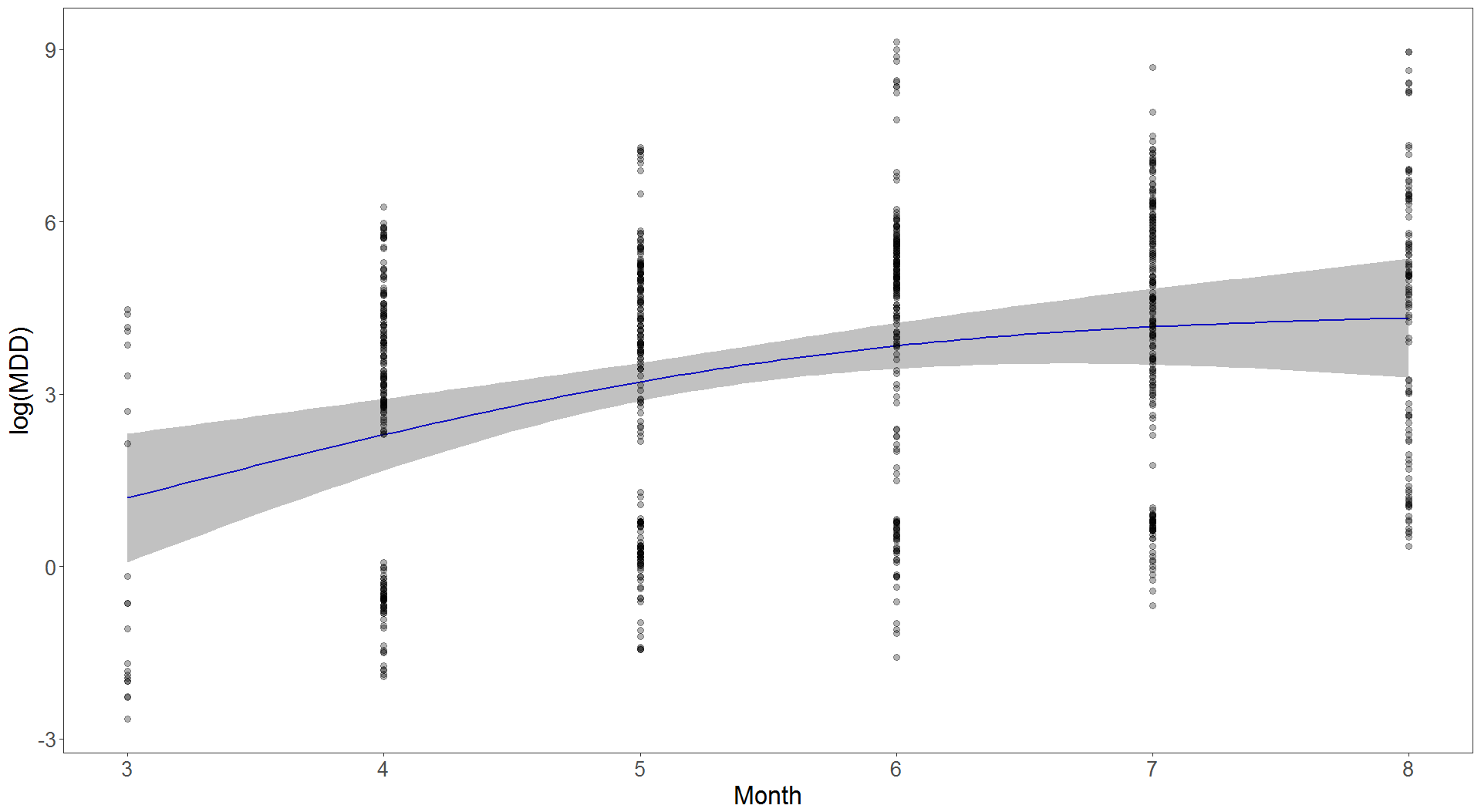

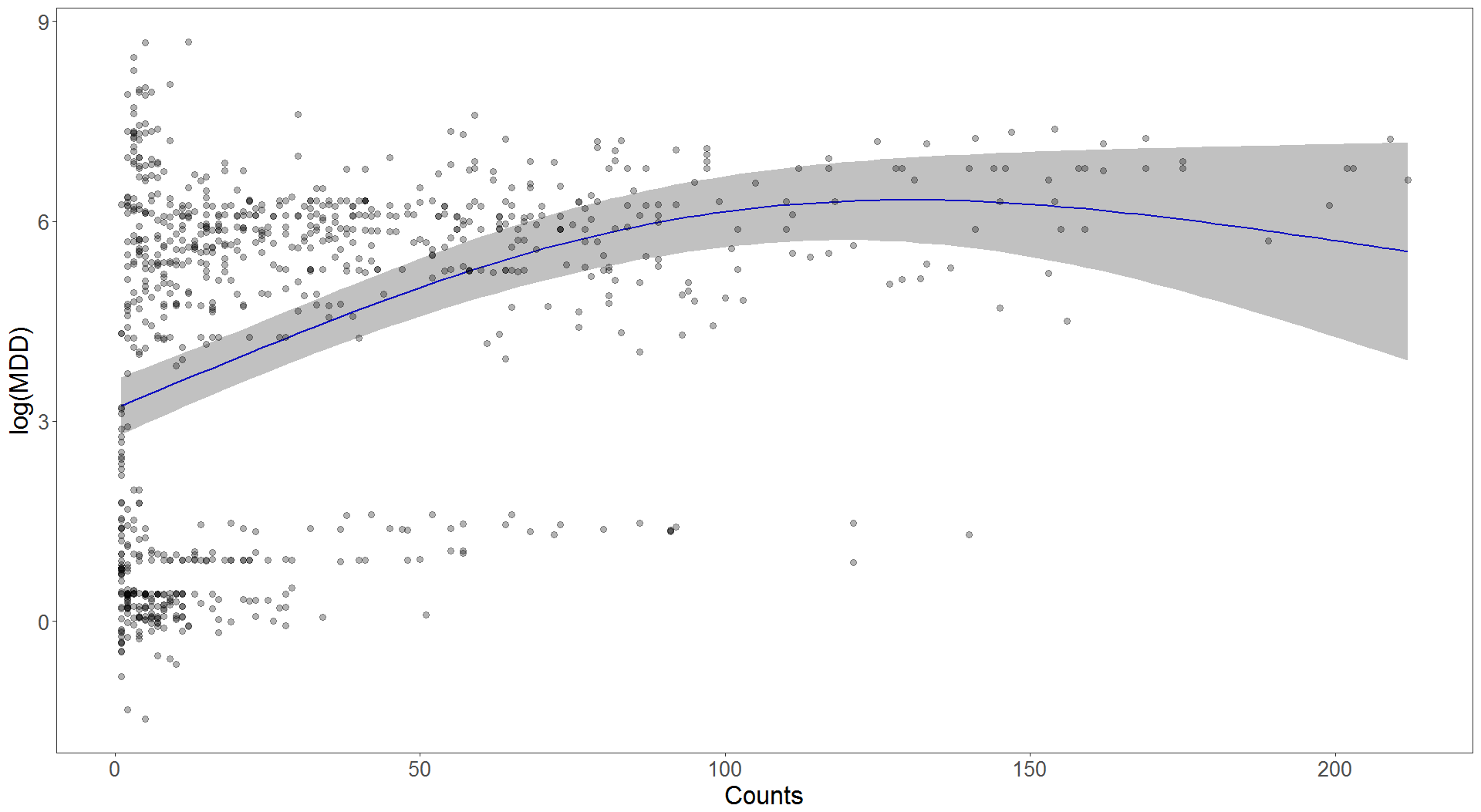

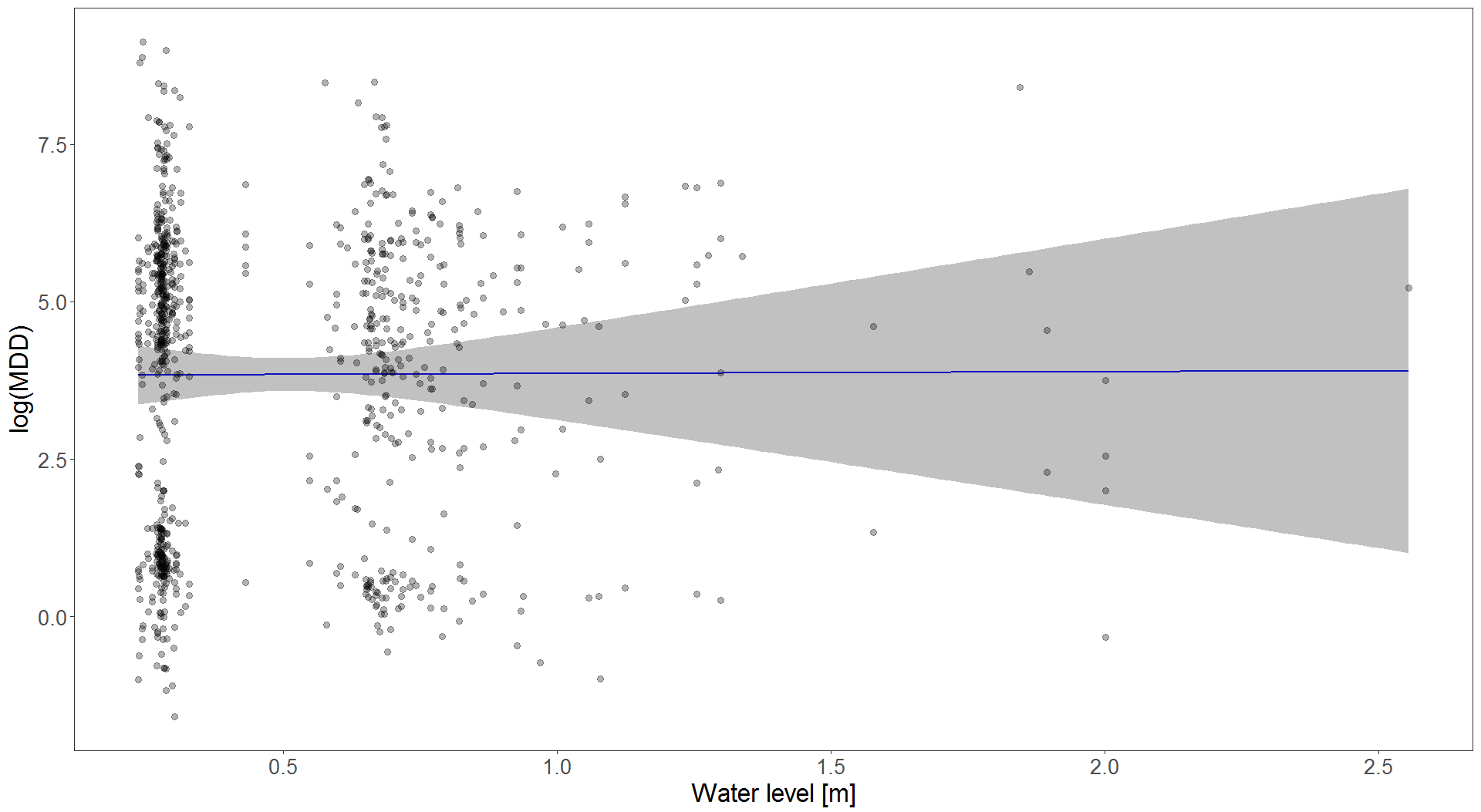


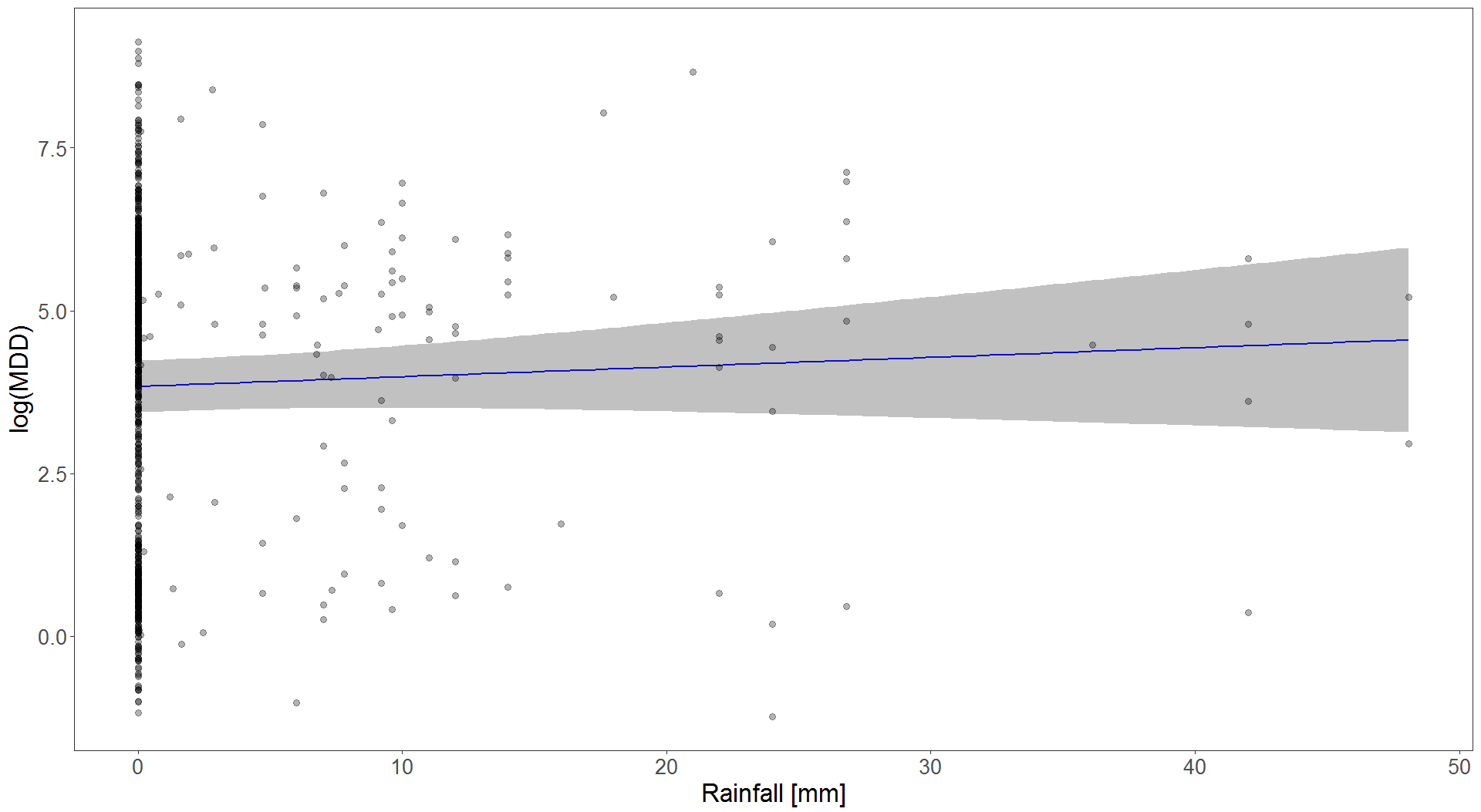


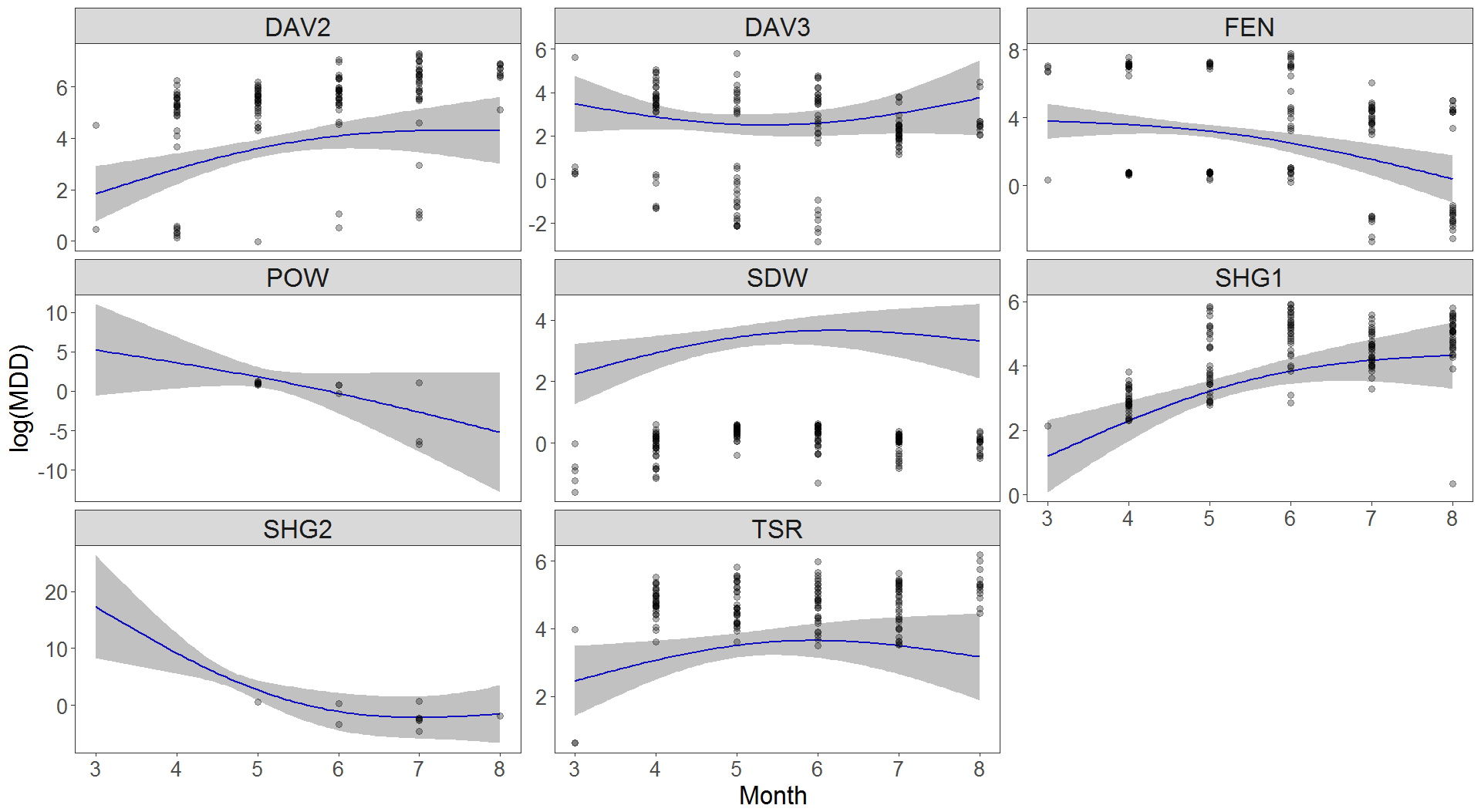


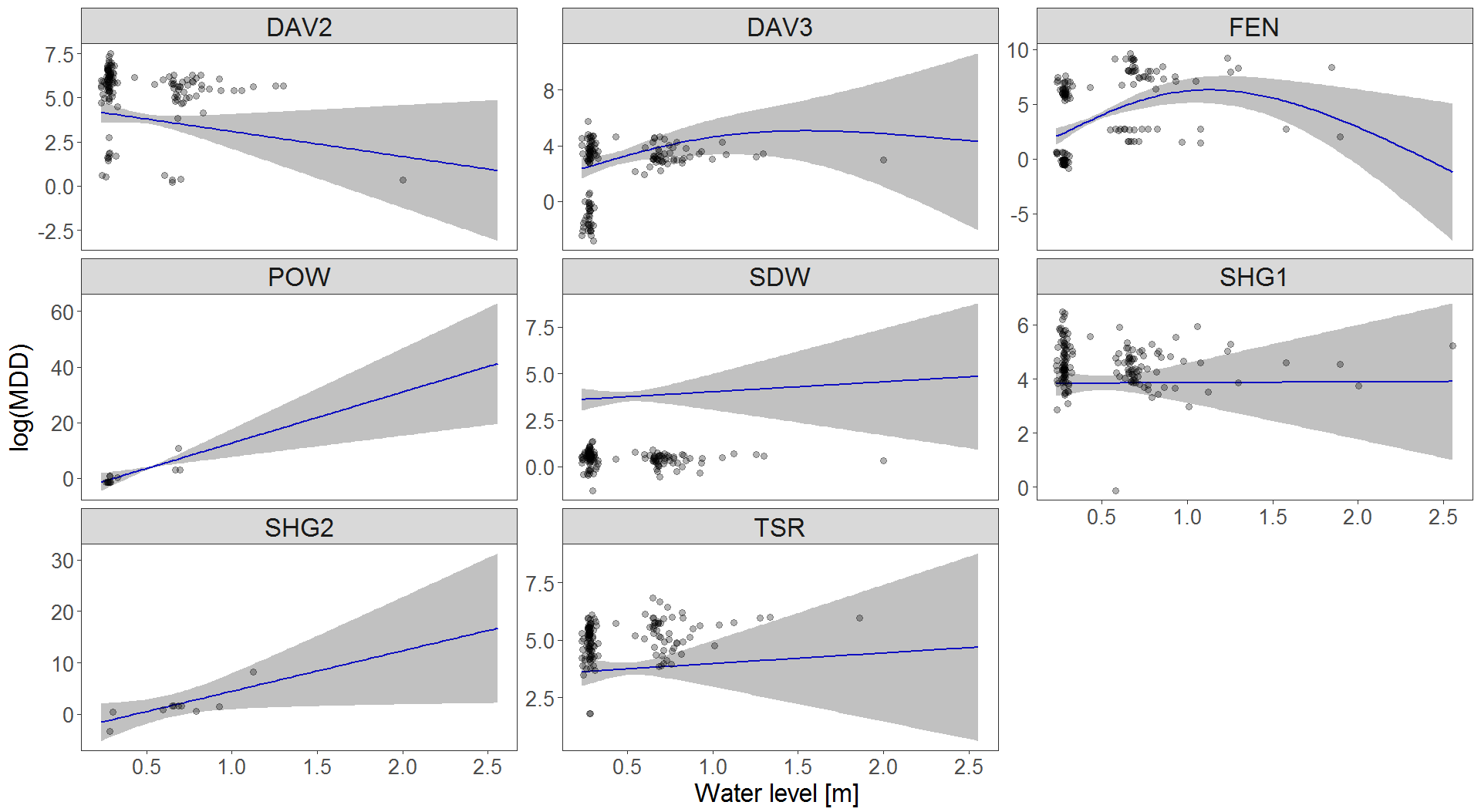


# Appendix 4: cumulative daily movements

Model coefficients (table) and predicted responses (plots) of Generalized Additive Model of the association between cumulative daily movements in response to month, water level, rainfall, and total counts with an interaction term between individual platypuses and month and water level.

| Parametric coefficients: | Estimate | S.E. | t | P |
| --- | --- | --- | --- | --- |
| Intercept | 0.75 | 0.01 | 53.62 | <0.001 |

| Smooth terms: | Edf | Df | F | P |
| --- | --- | --- | --- | --- |
| Water level [m] | 1.86 | 1.98 | 10.91 | 0.000 |
| Rainfall [mm] | 1.00 | 1.00 | 0.01 | 0.914 |
| Counts | 1.98 | 2.00 | 399.45 | <0.001 |
| Month | 1.00 | 1.00 | 2.91 | 0.088 |
| Wlevel:DAV2 | 1.00 | 1.00 | 12.85 | 0.000 |
| Wlevel:DAV3 | 1.90 | 1.99 | 9.21 | 0.000 |
| Wlevel:FEN | 1.00 | 1.00 | 11.51 | 0.001 |
| Wlevel:POW | 0.00 | 0.00 | 0.66 | 0.999 |
| Wlevel:SDW | 1.00 | 1.00 | 12.42 | 0.000 |
| Wlevel:SHG1 | 1.00 | 1.00 | 11.86 | 0.001 |
| Wlevel:SHG2 | 1.00 | 1.00 | 14.56 | 0.000 |
| Wlevel:TSR | 1.00 | 1.00 | 12.52 | 0.000 |
| Month:DAV2 | 0.00 | 0.00 | 0.27 | 0.994 |
| Month:DAV3 | 1.95 | 2.00 | 9.50 | 0.000 |
| Month:FEN | 1.00 | 1.00 | 7.99 | 0.005 |
| Month:POW | 1.92 | 1.99 | 9.66 | 0.000 |
| Month:SDW | 1.87 | 1.98 | 3.57 | 0.034 |
| Month:SHG1 | 1.96 | 2.00 | 12.97 | 0.000 |
| Month:SHG2 | 1.33 | 1.54 | 13.16 | 0.001 |
| Month:TSR | 1.56 | 1.81 | 1.08 | 0.429 |

R-sq.(adj) = 0.576 Deviance explained = 58.9%, -ML = 441.31 Scale est. = 0.16265 n = 855


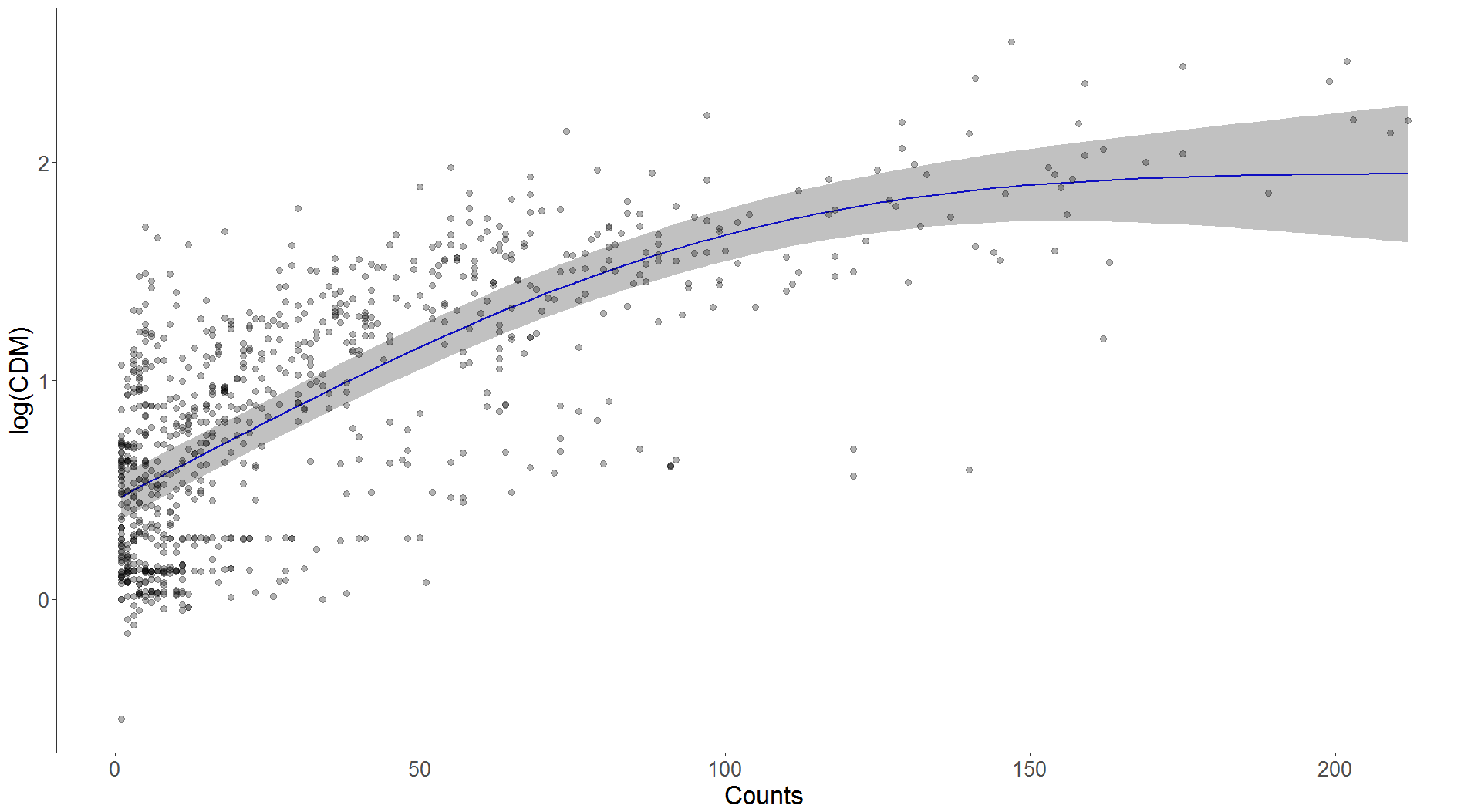

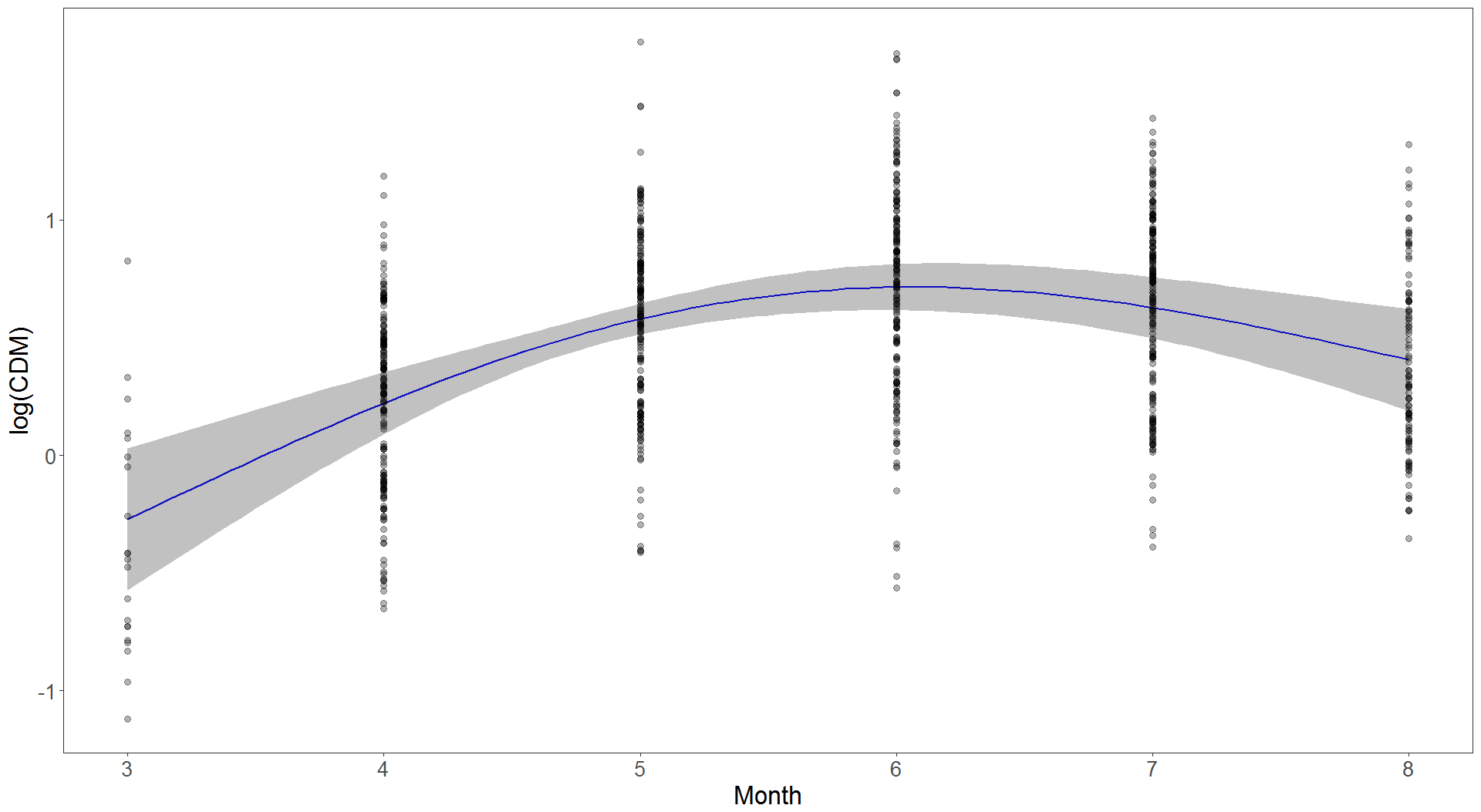


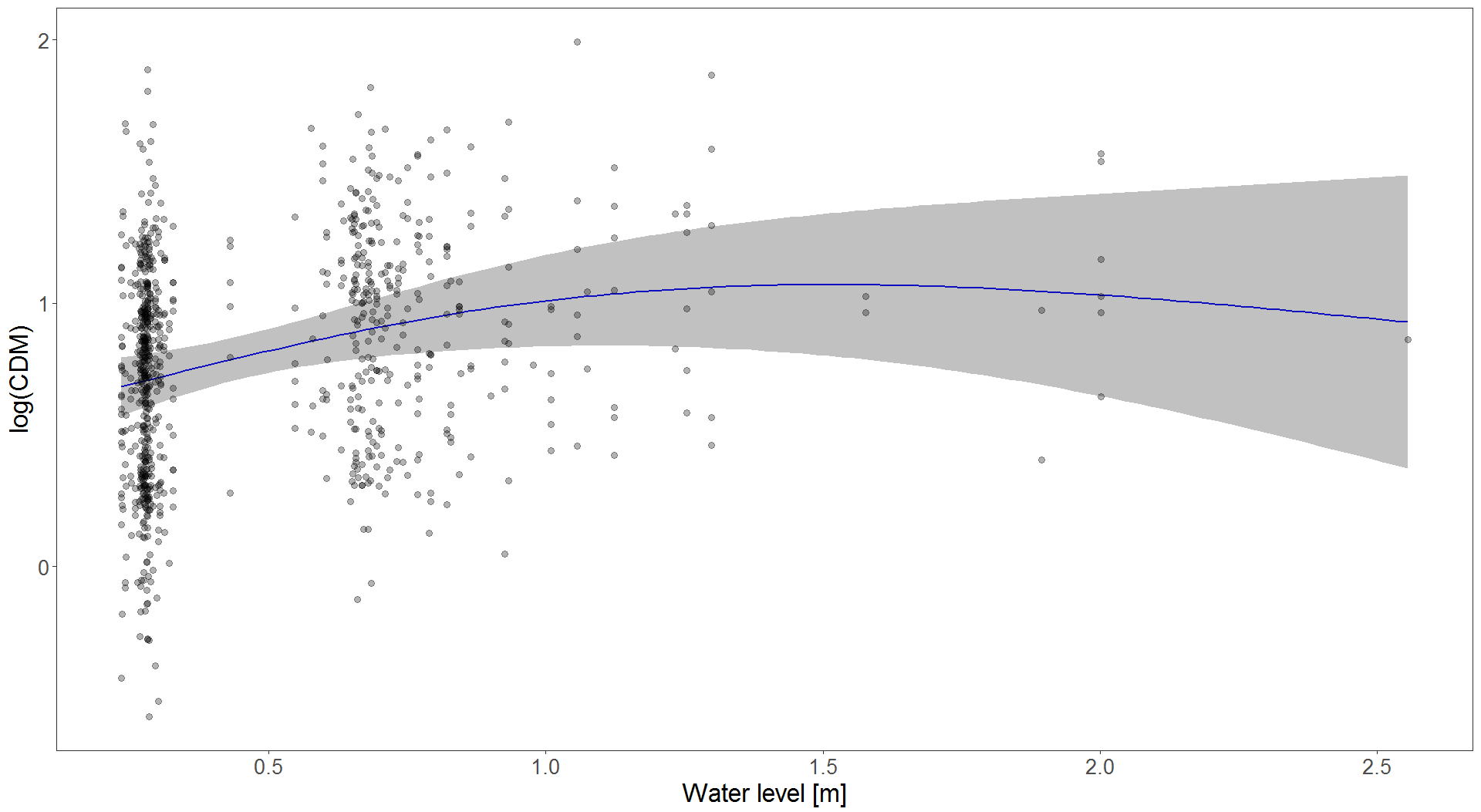


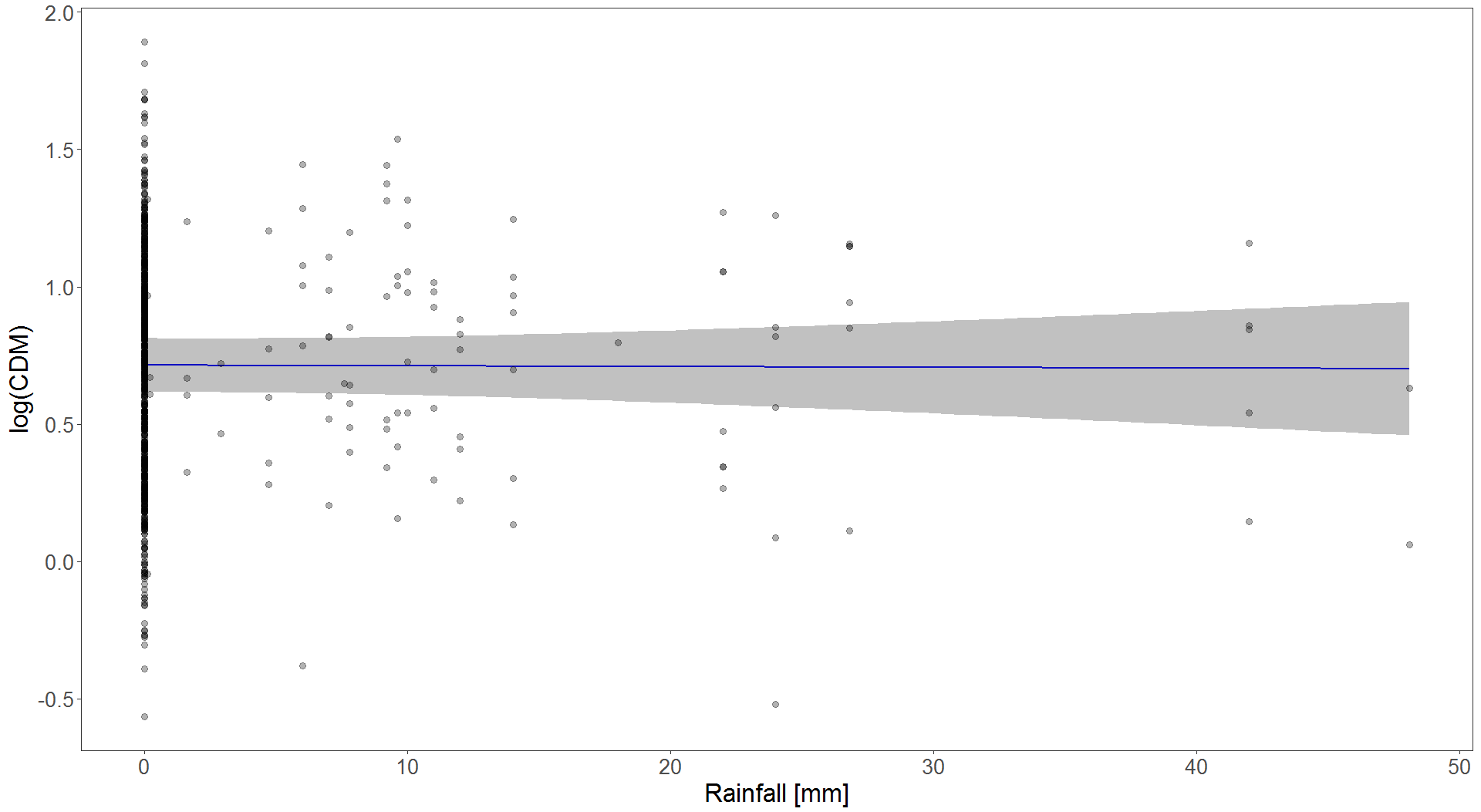


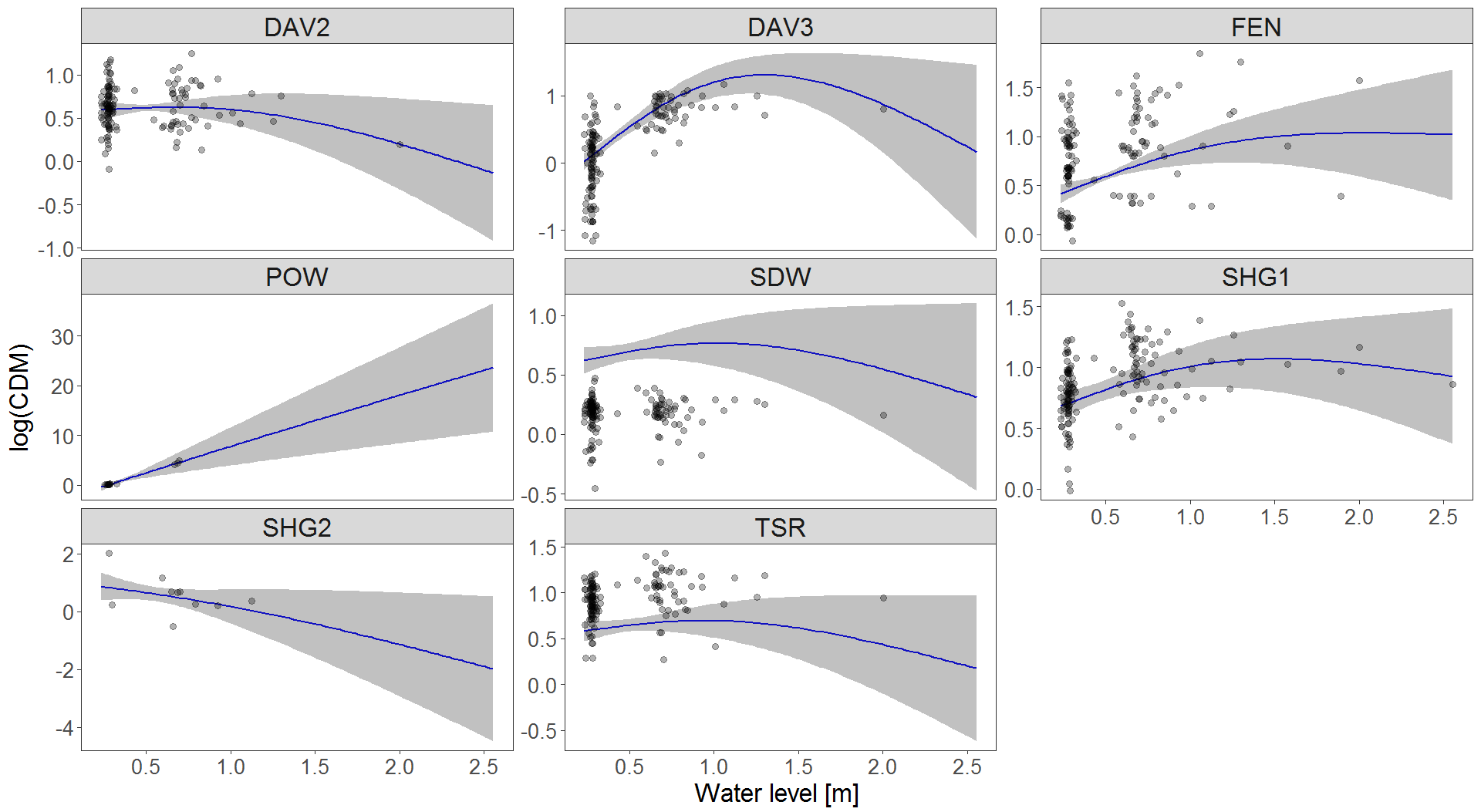

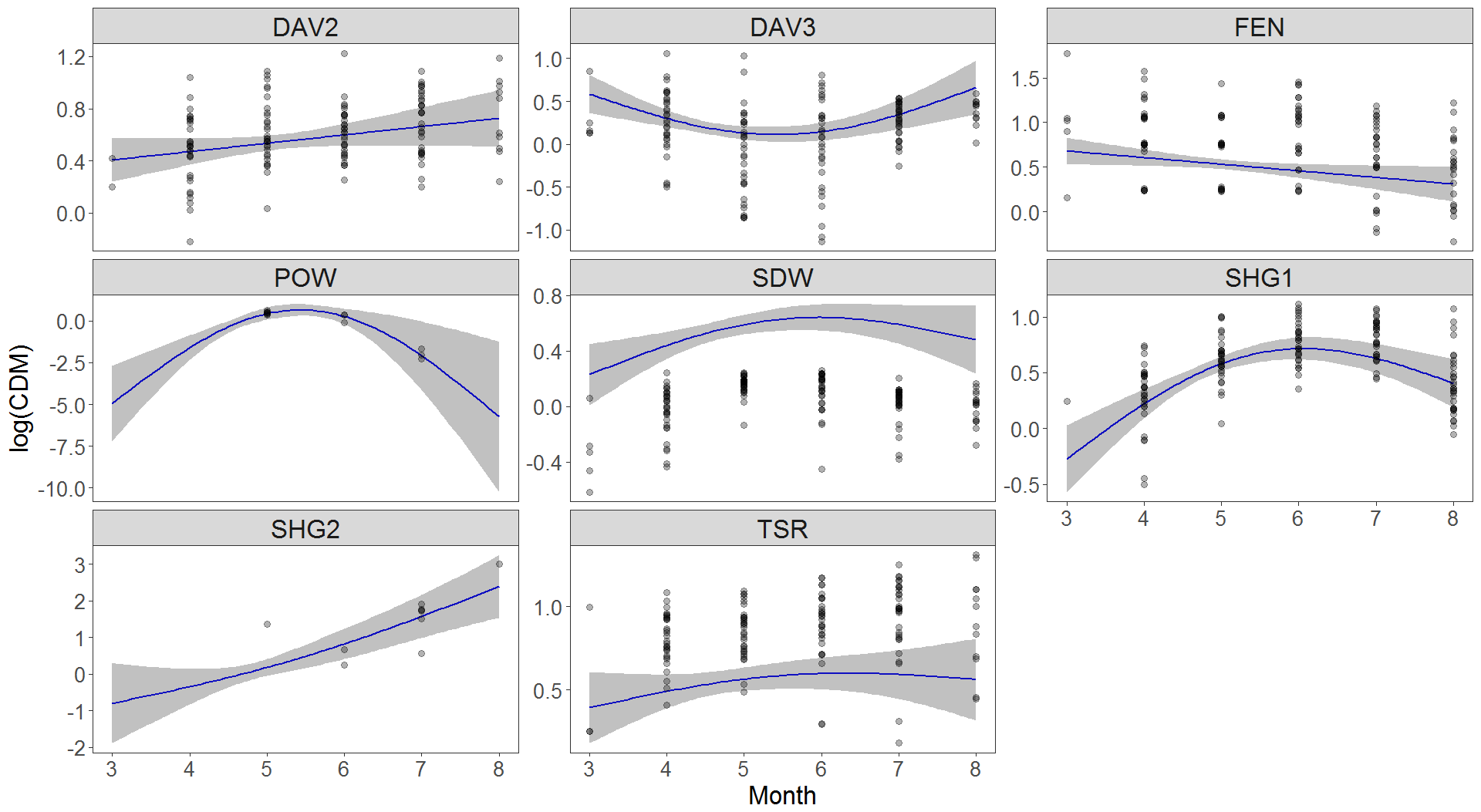

Supplement: Supplementary file 1 — Supplementary information [file 41598_2018_23461_MOESM1_ESM.docx]
